# Novel gene *Sen2* conferring broad-spectrum resistance to *Synchytrium endobioticum* mapped to potato chromosome XI

**DOI:** 10.1007/s00122-018-3154-y

**Published:** 2018-08-09

**Authors:** Jarosław Plich, Jarosław Przetakiewicz, Jadwiga Śliwka, Bogdan Flis, Iwona Wasilewicz-Flis, Ewa Zimnoch-Guzowska

**Affiliations:** 1 Plant Breeding and Acclimatization Institute – National Research Institute (IHAR-PIB), Młochów Research Center, Platanowa 19, 05-831 Młochów, Poland; 2 Plant Breeding and Acclimatization Institute – National Research Institute (IHAR-PIB), 05-870 Radzików, Błonie, Poland

## Abstract

**Abstract:**

***Key message Sen2*** **gene for potato wart resistance, located on chromosome XI in a locus distinct from** ***Sen1*** **, provides resistance against eight wart pathotypes, including the virulent ones important in Europe.**

**Abstract:**

*Synchytrium endobioticum* causes potato wart disease imposing severe losses in potato production, and as a quarantine pathogen in many countries, it results in lost trade markets and land for potato cultivation. The resistance to *S. endobioticum* pathotype 1(D1) is widespread in potato cultivars but new virulent pathotypes appear and the problem re-emerges. To characterize and map a new gene for resistance to potato wart, we used diploid F1 potato population from a cross of potato clone resistant to *S. endobioticum* pathotype 1(D1) and virulent pathotypes: 2(G1), 6(O1), 8(F1), 18(T1), 2(Ch1), 3(M1) and 39(P1) with a potato clone resistant to pathotype 1(D1) only. The 176 progeny clones were tested for resistance to eight wart pathotypes with a modified Glynne–Lemmerzahl method. Bimodal distributions and co-segregation of resistance in the population show that a single resistance gene, *Sen2*, underlies the resistance to eight pathotypes. Resistance to pathotype 1(D1) was additionally conferred by the locus *Sen1* inherited from both parents. *Sen2* was mapped to chromosome XI using DArTseq markers. The genetic and physical distances between *Sen1* and *Sen2* loci were indirectly estimated at 63 cM and 32 Mbp, respectively. We developed PCR markers co-segregating with the *Sen2* locus that can be applied in marker-assisted selection of potatoes resistant to eight important pathotypes of *S. endobioticum*. Wide spectrum of the *Sen2* resistance may be an indication of durability which can be enhanced by the pyramiding of the *Sen2* and *Sen1* loci as in 61 clones selected within this study.

**Electronic supplementary material:**

The online version of this article (10.1007/s00122-018-3154-y) contains supplementary material, which is available to authorized users.

## Introduction

*Synchytrium endobioticum* (Schilberszky) Percival is the causal agent of potato (*Solanum tuberosum* L.) wart disease. The pathogen originates from the Andean region of South America and has been spread throughout the world with the infected or contaminated potato tubers. The presence of potato wart has been reported in Asia, Africa, Europe, Oceania, North America and South America (Baayen et al. 2006; Obidiegwu et al. 2014). Currently, *S. endobioticum* is considered the most important quarantine pathogen of potato in almost all countries where potatoes are grown. It causes serious yield losses as well as a long-term soil contamination, since the winter sporangia are able to survive in the soil more than four decades (Przetakiewicz 2015a). Chemical control of the pathogen in field conditions is not possible. The disease can be restricted only by quarantine and phytosanitary measures and cultivation of resistant cultivars (Ballvora et al. 2011).

The pathotype 1 synonymous to 1(D1) of *S. endobioticum* was the first one that has occurred in Europe in the nineteenth century. The conventional breeding programs were successful in controlling potato wart disease through the development of resistant cultivars (probably with the *Sen1* gene), early in the twentieth century. At that time, it was believed that *S. endobioticum* is homogenous in terms of its parasitic proprieties, and a good level of control can be achieved by using existing resistant potato cultivars. However, from 1940s, new pathotypes of *S. endobioticum* began to spread. These virulent pathotypes overcame the resistance that was sufficient against pathotype 1(D1), making the control of the disease ineffective. According to the hypothesis proposed by Malec (1974), the virulent pathotypes emerged on the cultivars classified as resistant, but exhibiting incomplete resistance reaction (border genotypes). Up to date, about 40 pathotypes of *S. endobioticum* have been identified in Europe, among which 1(D1), 2(G1), 6(O1), 8(F1) and 18(T1) are considered as the most important and widely distributed (Baayen et al. 2006; Ballvora et al. 2011; Busse et al. 2017; Przetakiewicz 2014). There are also pathotypes with a narrow distribution but of a potential economic importance, for example the three virulent pathotypes of *S. endobioticum* named 2(Ch1), 3(M1) and 39(P) discovered in Poland (Malec 1981; Malinowska and Butrymowicz 2007; Przetakiewicz 2015b).

The genetics of potato resistance to *S. endobioticum* is still poorly understood (Ballvora et al. 2011). In the past, several models have been proposed to explain the genetic control of resistance to pathotype 1(D1). Initially, the studies on resistance to wart disease suggested combinations of two or more genes required for the resistance phenotype (Ross 1986). However, these studies were performed mainly on tetraploid potato clones with a tetrasomic inheritance, which could have been a serious impediment. Later it has been shown that a single gene *Sen1* determines the resistance to pathotype 1(D1) of *S. endobioticum* (Lellbach and Effmert 1990). Hehl et al. (1999) mapped the *Sen1* gene on potato chromosome XI, while Brugmans et al. (2006) reported the second resistance gene *Sen1*-*4* on potato chromosome IV. In three independent studies on resistance of tetraploid potato to the set of *S. endobioticum* pathotypes (1(D1) and three virulent pathotypes 2(G1), 6(O1), 18(T1)), a number of QRLs affecting potato wart resistance were identified on potato chromosomes: I, IX and XI (Ballvora et al. 2011), I, II, VI, VII, VIII, X and XI (Groth et al. 2013) and I, III, IV, VI, X, XI and XII (Obidiegwu et al. 2015). Only locus *Sen1/RSe*-*XIa*, which is located in similar position on potato chromosome XI as the gene *Sen1*, has significant effect on the potato wart resistance in all these three studies. However, in the studies of Ballvora et al. (2011) and Groth et al. (2013), this locus affects resistance only to the pathotype 1(D1), while in study of Obidiegwu et al. (2015) it affects resistance against the pathotype 1(D1) as well as the three virulent pathotypes: 2(G1), 6(O1) and 18(T1).

Identification and introduction into potato breeding pool of a highly effective sources of resistance against pathotype 1(D1) of *S. endobioticum* has allowed development of a number of resistant cultivars, which have significantly reduced the threat of this pathotype. Presently, the virulent pathotypes of the pathogen are spreading in different parts of the world and become a more and more important problem for potato growers. Unfortunately, in the case of virulent pathotypes, the sources of resistance with efficacy comparable with those for 1(D1) were not described yet, and the number of potato cultivars extremely resistant to these pathotypes is limited. In this work, a diploid potato interspecific hybrid highly resistant to *S. endobioticum* is described. The examined clone was simultaneously resistant to pathotypes: 1(D1), the four most important in Western Europe: 2(G1), 6(O1), 8(F1) and 18(T1), as well as the three pathotypes found in Poland: 2(Ch1), 3(M1) and 39(P) (Jakuczun et al. 2013). The goal of this study was to determine inheritance of resistance against eight pathotypes of *S. endobioticum* using an unselected diploid potato progeny of the resistant clone, and to map genetic factors responsible for the resistance. We identified a new major locus, named *Sen2*, which provided a high level of resistance simultaneously to the applied eight pathotypes of *S. endobioticum*. The locus *Sen2* was mapped to potato chromosome XI, at different localization than the locus *Sen1*. Markers for marker-assisted selection (MAS) of individuals which possess this gene were also developed.

## Materials and methods

### Plant material

A diploid F1 mapping population SEN 12-01 (176 individuals) was developed by crossing the potato clones DG 97-264 (seed parent, P1) and DG 97-1805 (pollen parent, P2). The clone DG 97-264 was extremely resistant to the pathotype 1(D1) as well as to the seven virulent pathotypes (2(G1), 6(O1), 8(F1), 18(T1), 2(Ch1), 3(M1) and 39(P1)) of *S. endobioticum* (Jakuczun et al. 2013). The clone DG 97-1805 was resistant to the pathotype 1(D1), but susceptible to the seven virulent ones. Both parental clones were bred in IHAR-PIB, Młochów Research Center, Poland, and both were complex interspecific hybrids having *Solanum tuberosum*, *S. chacoense*, *S. yungasense*, *S. gourlayi*, *S. acaule*, *S. demissum*, *S. verrucosum*, *S. microdontum* and *S. phureja* in their pedigrees. All progeny clones, parents and the standard susceptible potato cultivars Evora, Deodara and Tomensa were field propagated to provide tubers for the resistance tests.

### Assessments of resistance against *S. endobioticum*

All resistance tests were performed in Laboratory of Quarantine Organisms, Department of Plant Pathology, IHAR-PIB Radzików. Resistances against seven isolates representing the reference virulent pathotypes 2(G1), 6(O1), 8(F1), 18(T1), 2(Ch1), 3(M1) and 39(P1) were assessed in three consecutive years (2014–2016), while the resistance against an isolate of the pathotype 1(D1) was tested in 2 years (2015 and 2016). List and origins of the applied isolates are shown in Table S1 (Online Resource 1). Resistance tests were performed about 4 months after harvest and storage at temperature 4–8 °C. All 176 SEN 12-01 progeny clones and the parental clones were inoculated with the eight isolates of *S. endobioticum*. The total number of tubers of clones from SEN 12-01 progeny tested in this experiment was 58 261. The number of tubers tested in individual years of experiment was, dependent on availability of tuber material, 12,500, 31,815 and 13,946 tubers in 2014, 2015 and 2016, respectively. The mean numbers of tubers from each progeny clone tested with the particular wart pathotype during whole experiment varied from 38.8 up to 41.9. The detailed information about mean numbers and ranges of tubers tested with each pathotype in 3 years of experiment is shown in Table S2 (Online Resource 2). Resistance was tested according to Glynne–Lemmerzahl method modified by Przetakiewicz (2008). Before the tests, the tubers were washed, dried and incubated at room temperature in dark until the sprouts reached the length of 0.5–2 mm. Then, on the whole tubers, around the sprout a warm vaseline ring was made around the sprout using a syringe. The tubers were cooled at 4 °C for a few minutes or overnight so that the vaseline solidified, and subsequently the water tightness of the ring was checked. At inoculation, the tubers were placed in plastic boxes lined with irrigation mats, and the vaseline rings were filled with tap water. In each plastic box, along with tubers of the tested genotypes, at least five tubers of one of extremely susceptible standard cultivars (Evora, Tomensa or Deodara) were included as a control of tests conditions. Fresh warts were cut into pieces (about 2 g) and placed directly into the rings. The tubers were incubated for 48 h at 10 °C. After 24 h, the warts were moved from one tuber to another to increase the likelihood of infection. After this period, the warts and rings were removed and the tubers were treated with copper oxychloride. Subsequently, the tubers were incubated in boxes covered with a lid at 12/22 °C (alternately 12/12 h) and sprayed regularly with water. Although the first symptoms of infection were visible after 9 days of incubation, the first assessment was carried out 5 days later because some late necroses and very late necroses are recognizable after 2 weeks of incubation. The resistance was scored in 1–5 scale, where 1 is extremely resistant, 2 is resistant, 3 is weakly resistant, 4 is slightly susceptible and 5 is extremely susceptible (Przetakiewicz 2009; Flath et al. 2014). The tubers scored in ranks 3, 4 and 5 were incubated for additional 2 weeks in plastic boxes, covered with a thin layer of moist sand at 16 °C in darkness. This cover mixture was moistened with water every second or third day during the incubation period. The final reaction of these sprouts was evaluated after 4 weeks of incubation and scored as 3, 4 or 5. Since the differentiation between the scores 3 and 4 could be difficult, thin tissue sections were inspected under the microscope for the presence of winter sporangia. If winter sporangia were present, the sprouts were scored as 4 (slightly susceptible).

For each potato clone/cultivar, a mean score was calculated from the individual scores of all tubers tested during whole experiment. For genetic mapping, the resistance to *S. endobioticum* was treated as a qualitative trait, and individuals from SEN 12-01 were categorized into two classes: susceptible or resistant. Potato clones were classified as resistant when the mean resistance score was ≤ 3.0, while clones with the mean score > 3.0 were susceptible. The fit of segregation to the expected ratio was verified by the Chi-square test. One-way ANOVAs were performed to confirm diversification of mean resistance levels of individuals from SEN 12-01 progeny and to estimate percentage of resistance variation which can be explained by the influence of ‘genotype’ factor. The separate ANOVAs were performed for the results obtained with each of the eight pathotypes of *S. endobioticum*. Multiple comparisons of mean values were performed using Tukey’s test, and the homogeneous groups were distinguished for the results of SEN 12-01 progeny clones. All statistical analyses were performed with the use of software: MS Excel and Statistica 12 (StatSoft Inc., Tulsa, OK, USA).

### Mapping of resistance factors

#### DNA isolation, DArTseq genotyping

Total genomic DNA was extracted from flash-frozen leaves of all examined potato genotypes using GenElute Plant Genomic DNA Miniprep Kit (Sigma-Aldrich, Germany) according to the supplier’s protocol. All progeny clones from the mapping population, along with the parental clones DG 97-264 and DG 97-1085, were genotyped by a genotyping-by-sequencing (GBS) approach (DArTseq™) at Diversity Arrays Technology Pty. Ltd. (Canberra, Australia) (http://www.diversityarrays.com/dart-application-dartseq). DArTseq™ represents a combination of a DArT complexity reduction methods and next-generation sequencing platforms (Sansaloni et al. 2011; Kilian et al. 2012; Raman et al. 2014). The DArTseq™ method generates a high number of presence and absence variant (PAV) and single-nucleotide polymorphism (SNP) markers that can be successfully used for genetic mapping (Li et al. 2015). In our study, only markers of PAV type (as binary score 0 or 1) were used for map construction. Genotyping yielded the total amount of 53,731 DArTseq markers. Those markers, which were not scored in parental clones or not segregating in progeny, were removed. Subsequently, markers with a total of more than 5% missing data points were removed from the dataset. The remaining four 704 DArTseq markers were used for grouping and genetic map calculation in JoinMap^®^ 4.1 (Van Oojien 2006). The grouping settings were the following: CP (cross-pollination/outbreeder full-sib family) population type, independence LOD (significance cutoff, LOD score > 3) as a grouping parameter. Evaluation and chromosome assignment of 12 linkage groups were carried out based on BLAST search of DArTseq sequences against a reference DM1-3 potato genome sequences (PGSC DM1-3 v 4.03). Linkage maps were calculated using a regression method (linkage significance cutoff, LOD score > 3) and the Haldane’s mapping function for the calculation of map distances. The common (P1 + P2) map of chromosome XI, containing 273 markers, was constructed using a maximum likelihood approach because using the regression mapping exceeded the software’s calculation power. The detailed settings applied for construction of maps are shown in Online Resource 3.

#### Development of PCR markers

Two approaches were applied to find the PCR markers suitable for MAS listed in Table S3 (Online Resource 4). In the first approach, the DArTseq markers closest to the resistance locus *Sen2*: 12461274ch11, 3728961ch11 and 12448821ch11 were chosen to develop the PCR markers. Genomic sequences of the DM1-3 reference potato genome (v.4.03) containing these DArTseq markers were retrieved and used for design of PCR markers: 1251_3, 2502_1, 2502_3, 5450_3 (Table S3) (patent application number P.424490).

The second approach was based on a hypothesis that the *Sen2* gene may be a typical resistance gene and a member of the NB-LRR gene family. NB-LRR genes in potato genome are organized in conserved clusters (Jupe et al. 2013), and putative NB-LRR genes closest to the estimated localization of the *Sen2* locus were used for markers design (markers Cl80.1 and 3333.4) and tested for the linkage with the gene. All these PCR primers were designed with the use of the program Primer3 (Untergasser et al. 2012; Koressaar and Remm 2007). Markers Nl25 and Nl27 were used in SEN 12-01 population for confirmation of the presence of locus *Sen1*. According to Hehl et al. (1999), these DNA markers are linked with the gene *Sen1* conferring resistance to pathotype 1(D1) of *S. endobioticum*. Additionally, marker BCH was used as a positive control of PCR reactions (Milczarek 2012).

All PCRs were performed in standard PCR reactions (20 µl of reaction mixture containing: 0.06 U/µl of DreamTaq DNA Polymerase (Thermo Scientific), 0.2 mM of each dNTPs, 2 mM of MgCl_2_, primers (1–2 µl of 10 µM solution), template DNA (1 ng/µl), water (to 20 µl)) with the use of thermocycler program: 93 °C—3 min., 35 × (93 °C—2 min, 58 or 60 °C—45 s, 72 °C—1.5 min), 72 °C—10 min). The PCR products were separated in a 1–2% agarose gel stained with ethidium bromide.

## Results

### Phenotypic tests

#### Resistance of parental clones and standard cultivars

The parental clone DG 97-264 was extremely resistant to *S. endobioticum,* with mean resistance against each tested pathotype equal to 1.0. The resistance of parental clone DG 97-1805 against pathotype 1(D1) was very high (mean score 1.0), while it was susceptible to the seven virulent pathotypes with the mean scores of resistance: 4.0, 4.0, 4.4, 4.4, 4.6, 4.0 and 4.5 for pathotypes 2(G1), 6(O1), 8(F1), 18(T1), 2(Ch1), 3(M1) and 39(P1), respectively. The standard cultivars were extremely susceptible to all pathotypes. The mean scores of Evora, Deodara and Tomensa were 5.0 in all cases apart from cultivar Tomensa scored with 4.5 when infected with pathotype 6(O1).

#### Resistance of progeny SEN 12-01

**Fig. 1:**
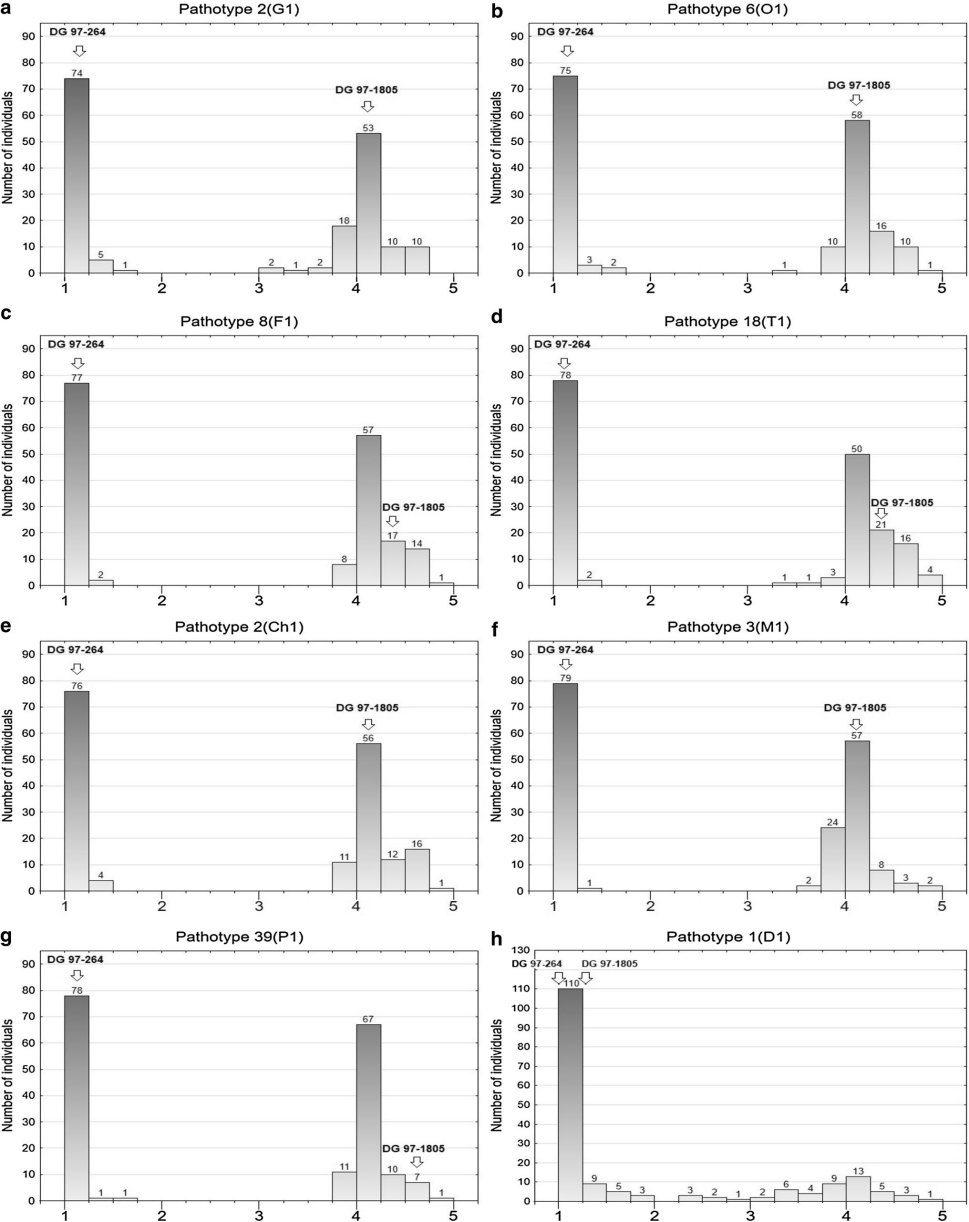
Frequency distributions of the mean score of resistance to eight pathotypes of *S. endobioticum* (**a** 2(G1), **b** 6(O1), **c** 8(F1), **d** 18(T1), **e** 2(Ch1), **f** 3(M1), **g** 39(P1), **h** 1(D1)) in a diploid F1 potato population SEN 12-01 (*N* = 176). X-axis: resistance level on 1–5 scale (where 1 is extremely resistant and 5 is extremely susceptible), y-axis: number of individuals. Resistance of parents (DG 97-264 and DG 97-1805) is indicated by arrows above the appropriate bars

One-way ANOVAs showed that progeny clones from population SEN 12-01 were diversified in terms of levels of resistance against the eight pathotypes of *S. endobioticum* (in each case *p* < 0.001). A majority of observed variation of resistance was explained by the plant genotype, as coefficients of determination *R*^2^ ascribed to this factor ranged from 0.96 to 0.99 depending on pathotype of *S. endobioticum* used in resistance tests. The distributions of resistance to seven virulent pathotypes were clearly bimodal, with the group of 80 highly resistant clones and a group of 96 susceptible ones (Fig. 1a–g). The same progeny clones were resistant to all virulent pathotypes of *S. endobioticum,* and there were no clones of intermediate resistance. The resistant clones were included to one homogenous group according to Tukey’s tests. The ratio of clones resistant and susceptible to virulent pathotypes of *S. endobioticum* in progeny SEN 12-01 was 1:1 (*χ*^2^ = 1.45; *p* = 0.23), which fits to the hypothesis of a single gene conferring the resistance. Of the 80 clones classified as resistant, 97.1% of tested tubers were scored as 1 (extremely resistant), 2.7% of tubers were scored as 2 (resistant) and 0.2% of tubers only were scored as 3 (weakly resistant). Rating 3 was observed in a few tubers of six resistant progeny clones.

Among resistant progeny, the clone SEN 12-01-113 expressed a different pattern of resistance, since it was resistant to the pathotypes 2(G1), 6(O1), 18(T1), 2(Ch1), 3(M1) and 39(P1), but susceptible to the pathotype 8(F1). This reaction for inoculation with the pathotype 8(F1) was observed in all 3 years of testing. Out of a total of 40 tubers of this clone inoculated with the pathotype 8(F1), 35 tubers were scored as 4, and five tubers were scored as 5. The resistance of SEN 12-01-113 to all remaining virulent pathotypes was very high (229 tubers scored as 1 and 11 scored as 2). Since susceptibility of this clone to the pathotype 8(F1) was the only exception, in further analysis this clone was regarded as a resistant one.

The distribution of resistance to the pathotype 1(D1) was also bimodal, but there was a strong overrepresentation of the resistant clones (Fig. 1h). The reaction of tested clones for inoculation with 1(D1) was more quantitative than in the case of the virulent pathotypes as indicated by the presence of clones with intermediate resistance. According to the adopted resistance threshold (≤ 3.0), 133 clones were resistant and 43 clones were susceptible to the pathotype 1(D1), and this ratio was consistent with 3:1 (*χ*^2^ = 0.03; *p* = 0.86). The resistant group included the same 80 individuals that had been assessed as resistant to the seven virulent pathotypes and 53 individuals that were susceptible to these pathotypes. For the 80 clones, 99.1% of their tubers challenged with 1(D1) were scored as 1, and 0.9% were scored as 2. For group of 53 clones resistant exclusively to 1(D1), the percentages of tubers scored as 1, 2 or 3 after inoculation with 1(D1) were 77.3, 14.5, and 5.2%, respectively. For eight clones from this group, 3.0% of tubers had the score 4 (weakly susceptible). The remaining 43 progeny clones were susceptible to all eight examined pathotypes of *S. endobioticum*.

The fact that all 80 genotypes resistant to the seven virulent pathotypes were present in the group of 133 clones resistant to 1(D1) indicates that the genetic factor that confers resistance to the virulent pathotypes also confers resistance to 1(D1). However, there is also at least one another independent factor conferring only resistance to 1(D1) in the progeny.

### Mapping of *Sen2* locus

**Fig. 2:**
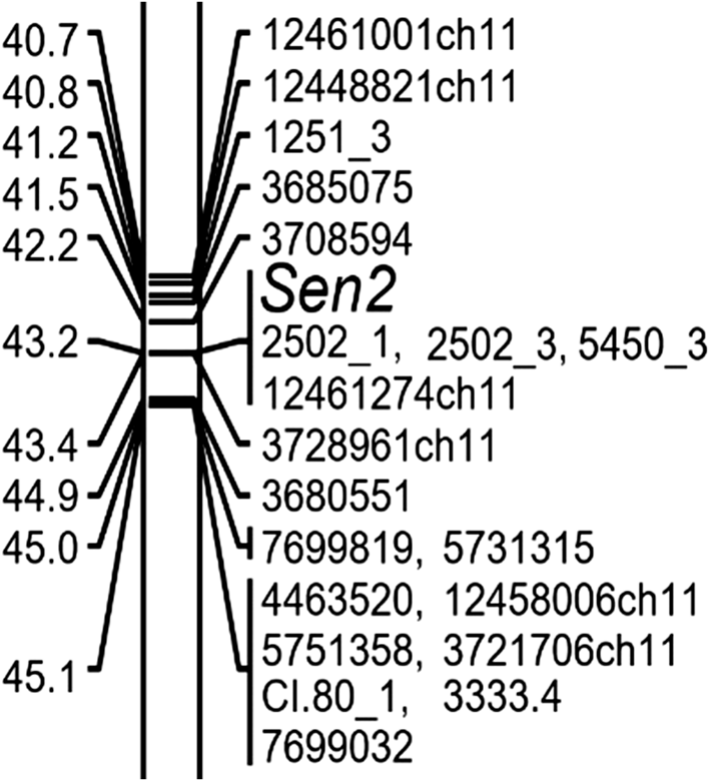
Fragment of genetic map of potato chromosome XI of resistant parent (P1) showing the position of locus *Sen2* for resistance to *S. endobioticum*. Whole map constructed of 95 DArTseq and six PCR markers, using 176 individuals of the population SEN12-01 and maximum likelihood algorithm in JoinMap^®^4.1 (Van Oojien 2006), is shown in Online Resource 3 (*Sen2* P1). Genetic distances (cM) on the left and marker/loci names on the right of the chromosome representation

The individuals of the SEN 12-01 progeny, along with their parental clones, were genotyped with the DArTseq™ method. For mapping of the genetic factors conferring potato wart resistance segregating in this progeny, 4704 DArTseq markers and two PCR markers (Nl25 and Nl27) linked with *Sen1* locus were used. After grouping of all markers, 12 linkage groups, corresponding to 12 potato chromosomes, were obtained (data not shown). A locus conferring the resistance to seven virulent pathotypes of *S. endobioticum*, named *Sen*2, was identified on chromosome XI; therefore, genetic maps of this chromosome were constructed. At first, a map of resistant parent DG 97-264 (P1) chromosome XI was constructed of 95 DArTseq markers originating from this parent. Using the preliminary mapping data, six PCR markers targeted at the *Sen2* locus were added to the map, and the resulting final map of length 73.4 cM is shown in Fig. 2 and in detail in Online Resource 3 (*Sen2* P1). The locus *Sen2* is located at 43.2 cM, co-segregating with DArTseq marker 12461274ch11 and PCR markers 2502_1, 2502_3 derived from it and a PCR marker 5450_3. The marker 5450_3 was designed on the basis of sequence of the marker 3728961ch11, which was located 0.2 cM from the locus *Sen2* in preliminary mapping. DArTseq marker 12461274ch11 nucleotide sequence was 69 bp long, and its best match in potato reference genome DM1-3 v.4.03 was at chr11:35055625..35055693 (PGSC0003DMG400032502, hypothetical gene of unknown function) (BLASTN 2.2.26, identity 95.65%, e value = 3e^−24^). However, the same BLAST search brought also nine other hits with identity > 90%, e values from 4e^−19^ to 1e^−23^, all located on chromosome XI within the range: 34.6–35.1 Mbp (data not shown).

**Fig. 3:**
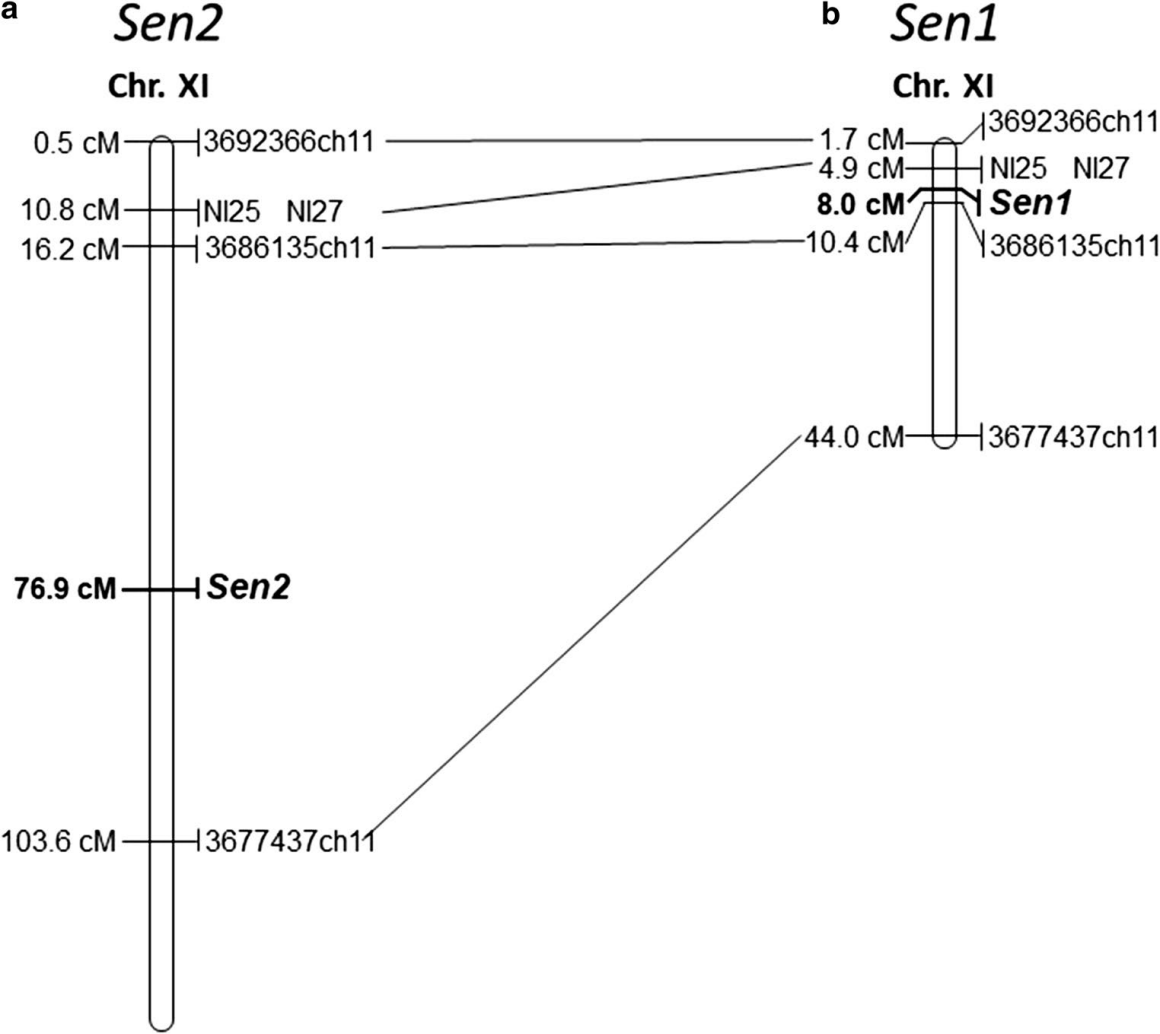
Positions of loci *Sen1* and *Sen2* for *S. endobioticum* resistance on the simplified genetic maps of chromosome XI developed using diploid F1 potato population SEN 12-01. Only five markers per map are shown, and the remaining ones are listed in the Online Resource 3. **a** Joined map (P1 + P2) of chromosome XI showing the position of *Sen2*, constructed of 273 markers, using 176 individuals and a maximum likelihood algorithm. **b** Joined map (P1 + P2) of chromosome XI showing the position of *Sen1*, constructed of 150 markers, using 96 individuals, regression mapping and Haldane’s mapping function. Maps were constructed using JoinMap^®^ 4.1 (Van Oojien 2006). Genetic distances (cM) on the left and marker/loci names on the right of the chromosome representations

Both parents of the SEN 12-01 progeny were resistant to the pathotype 1(D1) and had the Nl25 and Nl27 marker alleles indicating the presence of the locus *Sen1* (Fig. 3). In progeny SEN 12-01, the markers Nl25 and Nl27 co-segregated with each other and were present in 110 and absent in 66 individuals, which differs significantly from the expected 3:1 segregation ratio (*χ*^2^ = 14.7; *p* = 0.0001). Due to the fact that phenotypic effects of locus *Sen1* and the gene *Sen2* on resistance to pathotype 1(D1) were indistinguishable, the localization of the locus *Sen1* on the same map together with *Sen2* was impossible. Therefore, to show the position of locus *Sen1* in relation to *Sen2*, two more maps were constructed. Those markers, which were present on both of these maps, were arranged in similar orders (Online Resource 3, *Sen1* and *Sen2* (P1 + P2)).

The joined map of potato chromosome XI for both parents (Online Resource 3, *Sen2* (P1 + P2)) was constructed using 273 markers originating from P1 or P2 or from both parents, including the Nl25 and Nl27 markers and using the maximum likelihood algorithm. The *Sen2* (P1 + P2) map was 131.2 cM long. The locus *Sen2* was located at 76.9 cM of this map, while markers Nl25 and Nl27 were located at 10.8 cM and the genetic distance between locus *Sen2* and Nl markers was 66.1 cM (Online Resource 3, *Sen2* (P1 + P2); Fig. 3). Both Nl markers are annotated in the reference genome DM1-3, and while the position of Nl25 corresponded well with our genetic map, it was not the case for Nl27. Physical distance in DM1-3 reference genome between the Nl25 and DArTseq marker 12461274ch11 co-segregating with *Sen2* was 33.3 Mbp (Online Resource 3, *Sen2* (P1 + P2)).

To determine the position of the *Sen1* locus relative to the Nl25 and Nl27 markers, a joined P1 + P2 *Sen1* map was constructed (Online Resource 3, *Sen1*) using only 96 individuals of the SEN 12-01 population that did not have the *Sen2* resistance allele, i.e., that were susceptible to the virulent pathotypes of *S. endobioticum*. After this non-random selection, markers linked to the *Sen2* locus were not segregating among the chosen 96 individuals. In consequence, the *Sen1* map consisted of 150 markers (originating mostly from P2 or from both parents at the top of chromosome XI). Regression mapping and Haldane’s mapping function were applied. The map’s length was 46 cM. The locus *Sen1* was located at 8.0 cM on this map, while markers Nl25 and Nl27 were located at 4.9 cM (Online Resource 3, Sen1 (P1 + P2); Fig. 3) and the genetic distance between the *Sen1* locus and Nl markers was 3.1 cM. Physical distance in DM1-3 reference genome between Nl markers and the DArTseq marker 3686135ch11 flanking the *Sen1* locus from the opposite side than Nl markers was 1.3 Mbp (Online Resource 3, *Sen1* (P1 + P2)). By comparison with the reference genome DM1-3 of the locations of the markers flanking the *Sen1* locus (3687652ch11 at 1.636806 Mbp and 3686135ch11 at 3.078093 Mbp) with the location of a marker co-segregating with the *Sen2* (12461274ch11 at 35.055624 Mbp), we estimated the physical distance between the *Sen1* and *Sen2* loci to be between 32.0 and 33.4 Mbp (Online Resource 3). The genetic distance between loci *Sen1* and *Sen2* was estimated as 63 cM on the integrated map Sen2 (P1 + P2) (Online Resource 3) or between 33.5 and 35.1 cM on the maternal map (Online Resource 3 Sen2 (P1)), when we used Nl markers or maternal markers flanking Nl markers, respectively. The genetic distances were corrected by the distance between *Sen1* locus and Nl markers of 3.1 cM.

### Marker-assisted selection

To discriminate the presence of *Sen1* and *Sen2* in individuals of the SEN 12-01 progeny resistant to pathotype 1(D1) PCR, markers for both genes were applied in MAS. These markers were based on presence/absence polymorphism. In progeny SEN 12-01, 80 individuals resistant to eight pathotypes of *S. endobioticum* had markers 5450_3, 2502_1 and 2502_3 co-segregating with *Sen2*. Among these 80 progeny, 61 possessed also markers Nl25 and Nl27 linked to *Sen1*. In this group of clones, the number of recombinants between locus *Sen1* and both markers (Nl25 and Nl27) is unknown, since the presence or absence of the locus *Sen1* was masked by the phenotypic effect of *Sen2* locus. Markers Nl25 and Nl27 linked to *Sen1* were present also in 48 among 53 clones classified as susceptible to the virulent pathotypes but resistant to the 1(D1) pathotype, and in one clone susceptible to all pathotypes. Exemplary amplification products of PCR markers for MAS of potato clones with the *Sen1* (Nl27) and/or *Sen2* (5450_3) genes are shown in Fig. S1 (Online Resource 5).

The small variation of resistance to virulent wart pathotypes among SEN 12-01 progeny within groups: with *Sen2* and without *Sen2* gene was reflected by the percentages of variance in resistance that could be ascribed to the markers flanking the genes. The percentage of variance in resistance ascribed to markers 5450_3, 2502_1 and 2502_3 co-segregating with the *Sen2* varied in the range 95–98% depending on wart pathotype. In the case of the locus *Sen1* and the resistance to 1(D1), these percentages were smaller. For markers 5736102 and 3716309 flanking the *Sen1* locus (in the absence of *Sen2*), the respective percentages were 75 and 60%.

## Discussion

According to our best knowledge, this is the first work in which the resistance of unselected progeny against such broad spectrum of *S. endobioticum* pathotypes was studied. The resistance of 176 diploid potato clones against pathotype 1(D1) and seven virulent pathotypes 2(G1), 6(O1), 8(F1), 18(T1), 2(Ch1), 3(M1) and 39(P1) of *S. endobioticum* was examined. In total, more than 58 thousand tubers were tested. The results showed that parental clone DG 97-264 and 80 progeny clones were extremely resistant against all tested pathotypes of *S. endobioticum*. This very high resistance is provided by a newly identified broad-spectrum resistance gene *Sen2*, which was mapped on potato chromosome XI.

There was one unexpected observation concerning the progeny clone SEN 12-01-113, which was resistant to seven pathotypes of *S. endobioticum* but susceptible to the pathotype 8(F1). Since the same phenotype was observed during all 3 years of testing, we can exclude the hypothesis about accidental mixing of tested tubers. Such phenotypic results could suggest that resistances against particular virulent pathotypes of the pathogen could be governed by a set of closely linked resistance genes, and this individual is a recombinant. However, analysis of pattern of DArTseq markers in this individual (data not shown) showed that no recombination event was observed in the chromosomal region surrounding locus *Sen2* of clone SEN 12-01-113. The basis of this exceptional interaction between the pathotype 8(F1) of *S. endobioticum* and this potato genotype remains unknown, and to explain it, further studies are needed.

In our study, two resistance loci (*Sen2* and *Sen1*) were mapped. The first one, the gen *Sen2*, which provided resistance against eight pathotypes of *S. endobioticum*: 1(D1), 2(G1), 6(O1), 8(F1), 18(T1), 2(Ch1), 3(M1) and 39(P1), was mapped to 43.2 cM of potato chromosome XI of resistant parent DG 97-264. Based on the genomic localization of the nearest DArTseq markers 12448821ch11 and 12461274ch11, the most probable physical localization of the *Sen2* gene is in the region: chr11:33684487..35055624. In turn, the second locus *Sen1* provided resistance against pathotype 1(D1) only and was mapped at distal part of potato chromosome XI, 3.1 cM proximal from markers Nl25 (chr11:1804775..1808184) and the marker Nl27 (chr11:1340332..1347110 or chr11:11188091..11188558). The results of our mapping differ from those of Hehl et al. (1999), who mapped gene *Sen1* at the same region of potato chromosome XI, but 5.8 cM distal from Nl25 and Nl27 markers. This difference can result from various reasons, including differences in plant genotypes between studies, different methods of detection (hybridization or PCR), leading to detection of different *N*-related sequences. The physical distance between locus *Sen1* and newly identified gen *Sen2* was estimated at 32 Mbp.

The resistance gene *Sen2* is the first-identified major gene, which provides extreme resistance against broad spectrum of virulent pathotypes of *S. endobioticum*. According to ANOVAs, 95–98% (depending on pathotype) of variance in observed resistance of progeny SEN 12-01 can be ascribed to the presence of this gene (Table S4, Online Resource 6). Until now, only QRLs with smaller phenotypic effects on resistance against virulent pathotypes have been identified. These loci are located on almost all potato chromosomes (Ballvora et al. 2011; Groth et al. 2013; Obidiegwu et al. 2015). Some of these QRLs are located at various regions of potato chromosome XI, but explain only 6–22, 5–32 and 15% of observed variation in resistance against pathotypes 2(G1), 6(O1) and 18(T1), respectively (Groth et al. 2013). In study of Obidiegwu et al. (2015), locus *RSe*-*XIa* (synonymous to *Sen1*) located at potato chromosome XI also provided resistance against virulent pathotypes of *S. endobioticum* (2(G1), 6(O1) and 18(T1)), but the percentages of resistance variation explained by this locus were not given by the authors.

In almost all studies on potato resistance against *S. endobioticum* performed so far, the locus *Sen1/RSe*-*XIa* has been identified as the major contributor to potato wart resistance (Ballvora et al. 2011; Groth et al. 2013; Obidiegwu et al. 2015). In studies of Ballvora et al. (2011) and Groth et al. (2013), the resistance was considered as quantitative trait. In both these cases, the most important QRLs against potato wart were located in similar position at potato chromosome XI as gene *Sen1* and provided race-specific resistance against 1(D1) pathotype. In the study of Obidiegwu et al. (2015), the most important QRL for potato wart resistance was at locus *Sen1/RSe*-*XIa*. In contrary to other studies, the presence of this locus enhanced resistance not only against the pathotype 1(D1), but also against three virulent pathotypes. However, to achieve phenotypic effect on potato wart resistance, the presence of additional modifier genes was required (Obidiegwu et al. 2015). In our study, the locus *Sen1* provided resistance only to 1(D1). The percent of resistance variance which could be ascribed to markers 5736102 and 3716309 flanking *Sen1* locus (Online Resource 3, *Sen1* (P1 + P2)) equaled 75 and 60%, respectively. In 80 individuals resistant to all pathotypes, the phenotypic effect of locus *Sen1* was masked by the effect of the gene *Sen2*. However, 53 individuals were found to be susceptible to seven virulent pathotypes but usually extremely resistant to 1(D1). Only a few of these clones showed intermediate phenotypes (mean score 1.5–3.0) (Fig. 1h). In case of eight of these individuals, some of their tubers challenged with the pathotype 1(D1) were scored as 4. According to the adopted arbitrary limit (the mean score < 3.0), these clones were classified as resistant. But, according to qualitative criterion proposed by other protocols (even one tuber scored for 4 or 5 means susceptibility), all these clones should be regarded as susceptible (Przetakiewicz 2009). Only in two among these eight clones, the markers Nl25 and Nl27 (linked with the *Sen1* locus) were absent, which can suggest the lack of the *Sen1* locus.

The locus *Sen1/RSe-XIa* is probably the most common, but not only, genetic factor providing resistance to pathotype 1(D1) of *S. endobioticum* present in the modern gene pool of cultivated potato. The markers Nl25 and/or Nl27 linked with *Sen1* are present in many potato cultivars classified as resistant to pathotype 1(D1) (J. Plich—unpublished data). However, there are also some cultivars resistant to pathotype 1(D1) without these markers. These cultivars can be recombinants between the *Sen1* and Nl markers, or their resistance is provided by other genetic factors (e.g., locus *Sen1*-*4* located at potato chromosome IV).

Many potato cultivars possessing locus *Sen1/RSe-XIa* exhibit resistance to 1(D1), but the level of this resistance may range from extremely high to weak. It could be a result of the presence of different alleles at this locus or an influence of a specific genetic background (modifier genes). However, the influence of specific features of different isolates of the pathotype 1(D1) used for resistance testing cannot be also excluded. In the first decades of the twentieth century, there were thousands of foci of the pathotype 1(D1) across Europe and it is unlikely that all isolates of the pathogen were genetically identical. The classification of *S. endobioticum* to particular pathotypes is based on interaction with a set of differentials; therefore, some differences between isolates of the same pathotype can be unnoticed in this phenotypic assessment. Applying more accurate technics based on DNA sequence analysis indicates the low level of genetic diversity among different pathotypes of *S. endobioticum*, but the presence of some genetic variation between different isolates of 1(D1) pathotypes was observed (Busse et al. 2017; Bonants et al. 2015). Therefore, in future studies more emphasis should be put on identification of used isolates instead of description of pathotypes only. Isolates of *S. endobioticum* used in our study were multiplied from isolates used as a reference for pathotypes (Online Resource 1, Table S1). We assume that pathotypes 1(D1), 2(G1), 6(O1) and 18(T1) used in published studies (Ballvora et al. 2011; Groth et al. 2013; Obidiegwu et al. 2015) were from the same source (JKI, Germany).

Among presently available potato cultivars, a very few are extremely resistant to multiple pathotypes of potato wart (J. Przetakiewicz—personal communication). It is caused mainly by the low effectiveness of sources of resistance to virulent pathotypes of *S. endobioticum* available for potato breeders. For example, cultivar Panda (used as resistant parent in study of Groth et al. (2013)) is regarded as a donor of polygenic resistance, but only very few progeny clones from mapping population showed level of resistance as high as Panda (score 1) for some of virulent pathotype (Groth et al. 2013). Another major impediment in breeding of such cultivars is necessity of testing of resistance against multiple pathotypes, which is very laborious, expensive and time-consuming. The newly identified broad-spectrum resistance gene *Sen2* and the developed PCR marker co-segregating with this gene could help to omit both these obstacles. However, our study was performed on diploid potato clones, and the efficacy of the *Sen2* gene in providing extreme resistance against broad spectrum of *S. endobioticum* pathotypes should be confirmed also on tetraploid level of cultivated potatoes. Also usefulness of developed PCR markers should be verified on genetic backgrounds other than mapping population. Therefore, our next step will be introduction of *Sen2* into tetraploid potato breeding pool and using of PCR markers in a set of *2x* and *4x* potato clones/cultivars along with their phenotyping.

Although *Sen2* is a broad-spectrum resistance gene and the presence of 1(D1)-specific *Sen1* locus is not necessary to provide resistance to broad spectrum of *S. endobioticum* pathotypes, we recommend to combine both these genes to enhance the durability of potato wart resistance. Development of new potato cultivars durably resistant to many pathotypes of *S. endobioticum* is highly demanded, especially on the starch potato market. The specificity of starch potato production (high share of potato in crop rotation) highly increases the threat of occurrence of potato wart disease in the fields. The new potato cultivars with the broad-spectrum resistance against multiple pathotypes of *S. endobioticum* will help also to restrict further spreading of virulent pathotypes of the pathogen.

According to Ross (1986), resistance to *S. endobioticum* has been introduced into cultivated potato from various sources, including tetraploid potato cultivars as well as wild *Solanum* species. The origin of resistance examined in our study remains unrecognized, because the resistance donor (diploid potato clone DG 97-264) is a complex hybrid of various *Solanum* species including: *S. tuberosum*, *S. chacoense*, *S. yungasense*, *S. gourlayi*, *S. acaule*, *S. demissum*, *S. verrucosum*, *S. microdontum, S. phureja*. Among these species, *S. acaule* is mentioned by several authors as source of potato wart resistance (Van Soest 1983; Ross 1966, 1986; Ballvora et al. 2011) and therefore it could be a potential donor of *Sen2* gene. However, there are no direct evidences supporting this hypothesis.

### Author contribution statement

EZG, JP^1^, JŚ, BF conceived the project; JP^1^ multiplied plant material and provided tubers for phenotyping, prepared DNA samples for genotyping, developed PCR markers and performed PCR marker screening, analyzed the phenotypic and genotypic data, assisted in maps construction and wrote the manuscript; JP^2^ provided pathotypes of *S. endobioticum* and carried out all resistance tests; JŚ contributed to construction of genetic maps, data analyses and interpretation as well as manuscript writing, BF supervised the work, assisted with statistical analysis; IW-F contributed to identification of resistant parent and development of the mapping population; EZG directed the project and revised the manuscript; all authors have read and approved the final manuscript.

## Electronic supplementary material

Below is the link to the electronic supplementary material.

Supplementary material 1 (DOCX 25 kb)

Supplementary material 2 (DOCX 35 kb)

Supplementary material 3 (XLSX 52 kb)

Supplementary material 4 (DOCX 34 kb)

Supplementary material 5 (DOCX 327 kb)

Supplementary material 6 (DOCX 26 kb)
