# Supplementary material for: Novel gene Sen2 conferring broad-spectrum resistance to Synchytrium endobioticum mapped to potato chromosome XI
Source: Theor Appl Genet. 2018 Aug 9;131(11):2321–31. doi: 10.1007/s00122-018-3154-y (PMC6208938; doi:10.1007/s00122-018-3154-y)
Supplement: Supplementary file 1 — Supplementary material 1 (DOCX 25 kb) [file 122_2018_3154_MOESM1_ESM.docx]

**Table S1. Isolates of *S. endobioticum* pathotypes used in the study.**

| Pathotype | Isolate#/LOA# | Country of collection |
| --- | --- | --- |
| 1(D1) | #1/2007/D1 | Germany* |
| 2(G1) | #4/2005/G1 | Germany |
| 2(Ch1) | #2/2005/Ch1 | Poland** |
| 3(M1) | #2/2005/M1 | Poland |
| 6(O1) | #1/2007/O1 | Germany |
| 8(F1) | #3/2005/F1 | The Netherlands*** |
| 18(T1) | #27/2009/T1 | Germany |
| 39(P1) | #PL69/2009 | Poland |

* From the reference bank of Julius Kühn-Institut (JKI), Federal Research Centre for Cultivated Plants, Institute for Plant Protection in Field Crops and Grassland, Stahnsdorfer Damm 81, 14532 Kleinmachnow, Germany

** From Bydgoszcz Research Center, Department of Root Crops Production Technology, Laboratory for Quarantine Diseases and Pests of Potato, Plant Breeding and Acclimatization Institute-National Research Institute, Poland

*** From the Plant Protection Service, National Reference Laboratory, PO Box 9102, 6700 HC Wageningen, The Netherlands
