# Supplementary material for: Novel gene Sen2 conferring broad-spectrum resistance to Synchytrium endobioticum mapped to potato chromosome XI
Source: Theor Appl Genet. 2018 Aug 9;131(11):2321–31. doi: 10.1007/s00122-018-3154-y (PMC6208938; doi:10.1007/s00122-018-3154-y)
Supplement: Supplementary file 2 — Supplementary material 2 (DOCX 35 kb) [file 122_2018_3154_MOESM2_ESM.docx]

**Table S2. The mean numbers and ranges of tubers tested per clone with eight pathotypes of *S. endobioticum* of SEN 12-01 progeny in 2014 – 2016.**

| **Year** | **2014** | | | **2015** | | | **2016** | | |
| --- | --- | --- | --- | --- | --- | --- | --- | --- | --- |
| **Pathotype** | **N*** | **Number of tested tubers** | | **N** | **Number of tested tubers** | | **N** | **Number of tested tubers** | |
|  |  | mean | range |  | mean | range |  | mean | range |
| 1(D1) | - | - | - | 134 | 18.9 | 8 – 38 | 176 | 20.1 | 3 – 40 |
| 2(G1) | 172 | 10.4 | 3 – 20 | 176 | 22.2 | 5 – 38 | 176 | 8.8 | 2 – 27 |
| 2(Ch2) | 173 | 10.0 | 2 – 20 | 176 | 23.4 | 8 – 40 | 176 | 8.2 | 1 – 27 |
| 3(M1) | 173 | 10.0 | 2 – 20 | 176 | 23.1 | 8 – 40 | 176 | 8.5 | 2 – 25 |
| 6(O1) | 173 | 9.4 | 3 – 20 | 176 | 23.2 | 8 – 40 | 176 | 8.7 | 1 – 27 |
| 8(F1) | 172 | 10.2 | 2 – 21 | 176 | 23.3 | 8 – 40 | 176 | 8.5 | 1 – 27 |
| 18(T1) | 172 | 10.4 | 2 – 20 | 176 | 23.2 | 5 – 38 | 176 | 8.4 | 1 – 27 |
| 39(P1) | 172 | 9.7 | 2 – 20 | 176 | 23.3 | 8 – 40 | 176 | 8.8 | 2 – 27 |

* - N – number of tested clones from SEN 12-01 progeny
