# Supplementary material for: Novel gene Sen2 conferring broad-spectrum resistance to Synchytrium endobioticum mapped to potato chromosome XI
Source: Theor Appl Genet. 2018 Aug 9;131(11):2321–31. doi: 10.1007/s00122-018-3154-y (PMC6208938; doi:10.1007/s00122-018-3154-y)
Supplement: Supplementary file 4 — Supplementary material 4 (DOCX 34 kb) [file 122_2018_3154_MOESM4_ESM.docx]

**Table S3.** PCR primers used in this study.

| **Marker** | **Sequence** | **Name of the source sequence and position in the DM1-3 v4.03 reference genome (bp)** | **T^1^ (°C)** | **Product size (bp)** | **References** |
| --- | --- | --- | --- | --- | --- |
| **Nl25 F**  **Nl25 R** | TATTGTTAATCGTTACTCCCTC  AGAGTCGTTTTACCGACTCC | PGSC0003DMG401015682  chr11:1804775..1808184 | 58 | ~1400 | Gebhardt et al. 2006 |
| **Nl27 F**  **Nl27 R** | TAGAGAGCATTAAGAAGCTGC  TTTTGCCTACTCCCGGCATG | PGSC0003DMG403016979  chr11:1340332..1347110 or  PGSC0003DMG400000889 chr11:11188091..11188558 | 58 | ~ 1200 | Marczewski et al. 2001 |
| **1251_3 F**  **1251_3 R** | TGGAGAAACCATCCAATTCC GACGAAGGGGTGTCTCTTGA | 12448821ch11 chr11:33684487..33684554 | 60 | ~1300^2^ | This study |
| **2502_1 F**  **2502_1 R** | GCAGAGTTTCCACTGCTTCA TGATTCGAAATTCAACCACTATG | 12461274ch11 chr11:35055625..35055693 | 60 | ~290 | This study |
| **2502_3 F**  **2502_3 R** | Ccaactccacaactttttgct ccaactgattcgaaattcaac | 12461274ch11 chr11:35055625..35055693 | 60 | ~1500 | This study |
| **5450_3 F**  **5450_3 F** | ATGATCATTGATGGCAGCAG GGGCATTTTGGAGCATAAAA | 3728961ch11  chr11:34894172..34894240 or  chr11:34934800..34934868 | 58 | ~1046 | This study |
| **3334_3 F**  **3334_3 R** | Gcaccaagttcggatatgct  ggggccaccaataaaaagat | PGSC0003DMG400033334^3^  chr11:37644263..37650835 | 58 | ~1408 | This study |
| **Cl.80_1 F**  **Cl.80_3 R** | TTTGAAGCGAAGTGAGGTGC  aagactttcggtgccatgtg | RDC0001NLR0272^3^ chr11:35554214..35558468 | 58 | ~1159^2^ | This study |
| **BCH F**  **BCH R** | CATGACATAGTTTGAATTTTGAGTC  CGTTTGGCGCTGCCGTAAGTT | PGSC0003DMG400010169  chr03:43936158..43938595 | 58/60 | ~300 | Milczarek et al. 2012 |

^1^Annealing temperature, ^2^ markers linked with the *Sen2* gene in repulsion phase, ^3^ –potato NBS-LRR genes and their genomic addresses, according to Jupe et al. 2013.
