# Supplementary material for: Novel gene Sen2 conferring broad-spectrum resistance to Synchytrium endobioticum mapped to potato chromosome XI
Source: Theor Appl Genet. 2018 Aug 9;131(11):2321–31. doi: 10.1007/s00122-018-3154-y (PMC6208938; doi:10.1007/s00122-018-3154-y)
Supplement: Supplementary file 5 — Supplementary material 5 (DOCX 327 kb) [file 122_2018_3154_MOESM5_ESM.docx]

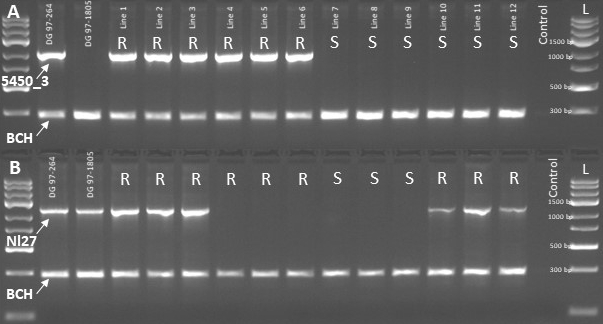


**Figure S1.** Products of amplification of PCR markers: 5450_3 which co-segregates with the gene *Sen2* (picture **A**) and Nl27 linked with the gene *Sen1* (picture **B**). In both cases the marker BCH was included as a PCR reaction control. Products of all markers are marked by arrows. DG 97-264 – parental clone of SEN 12-01 progeny resistant to all tested pathotypes of *S. endobioticum*, DG 97-1805 – parental clone of SEN 12-01 progeny resistant to pathotype 1(D1) and susceptible to virulent pathotypes of *S. endobioticum*. Line 1 – Line 12 – selected clones from SEN 12-01 progeny. Letters R and S mean resistant and susceptible, respectively (A: resistance / susceptibility to virulent pathotypes; B: to pathotype 1(D1) of *S. endobioticum*). L – DNA ladder (GeneRuler Express DNA Ladder, Thermo Scientific).
