# Supplementary material for: Novel gene Sen2 conferring broad-spectrum resistance to Synchytrium endobioticum mapped to potato chromosome XI
Source: Theor Appl Genet. 2018 Aug 9;131(11):2321–31. doi: 10.1007/s00122-018-3154-y (PMC6208938; doi:10.1007/s00122-018-3154-y)
Supplement: Supplementary file 6 — Supplementary material 6 (DOCX 26 kb) [file 122_2018_3154_MOESM6_ESM.docx]

**Table S3. Analysis of variance of resistance against particular pathotypes of *S. endobioticum* observed in SEN 12-01 progeny.**

| **Pathotype** | **df** | **Sum of squares** | **Mean of squares** | **P value** | **Corrected R^2^** |
| --- | --- | --- | --- | --- | --- |
| 1(D1) | 175 | 450.75 | 2.576 | *** | 0.966 |
| 2(G1) | 175 | 1223.08 | 6.989 | *** | 0.971 |
| 2(Ch2) | 175 | 1354.199 | 7.738 | *** | 0.976 |
| 3(M1) | 175 | 123.606 | 6.992 | *** | 0.970 |
| 6(O1) | 175 | 1280.191 | 7.316 | *** | 0.979 |
| 8(F1) | 175 | 1298.111 | 7.418 | *** | 0.988 |
| 18(T1) | 175 | 1357.646 | 7.758 | *** | 0.961 |
| 39(P1) | 175 | 1251.416 | 7.151 | *** | 0.991 |
